# Heparin effect in alveolar cells and macrophages in an acute lung injury model

**DOI:** 10.1186/2197-425X-3-S1-A570

**Published:** 2015-10-01

**Authors:** M Camprubí-Rimblas, R Guillamat-Prats, T Lebouvier, L Chimenti, M Iglesias, C Obiols, J Tijero, MN Gómez, C de Haro, L Blanch, A Artigas

**Affiliations:** Fundación Parc Taulí, Sabadell, Spain; Ciber de Enfermedades Respiratorias, Sabadell, Spain; Ponchaillou University Hospital, Surgical Intensive Care, Rennes, France; University Hospital of Mutua de Terrassa, University of Barcelona, Thoracic Surgery Service, Terrassa, Spain; Corporación Sanitaria y Universitaria Parc Taulí, Critical Care Center, Sabadell, Spain

## Introduction

Acute Lung Injury (ALI) and Acute Respiratory Distress Syndrome (ARDS) are characterized by a promptly release of proinflammatory mediators that downregulate natural anticoagulant mechanisms, initiate the coagulation system, impair fibrinolysis and produce the rupture of the endothelial and epithelial monolayer [1]. Inflammation of ALI/ARDS is initiated and strongly regulated by proinflammatory activation of macrophages, which lead to the recruitment of other inflammatory cells, producing alveolar cells injury, propagating the coagulant response and promoting the injury. Currently there is no effective treatment for this disease. Previous studies have presented the beneficial effect of anticoagulants, not only for their anticoagulation activity but also for their antiinflammatory action [2],[3].

## Objectives

To evaluate the effect of the treatment with heparin in human alveolar cells and alveolar macrophages after induce an injury, mimicking an *in-vitro* ALI.

## Methods

Human alveolar primary cells and alveolar macrophages from pulmonary biopsies were isolated and seeded. Sodium non-fractioned heparin (100 UI/ml) was administered to the cells after the induction of an injury with a pro-inflammatory stimulus (Cytomix: mix of TNFα, IL1β and IFNγ; 25 ng/ml or Lipopolysaccharide protein from *Escherichia coli* 055 : B5; 1000 ng/ml). The effect of heparin was assessed by the analysis of proinflammatory markers (IL12p40, iNOS, TNFα and IL-1β) and anti-inflammatory markers (IL10 and IL13), cell proliferation and permeability (measuring transmembrane resistance). Data are expressed as mean ± SEM (units are relative to the expression of control group). Statistical analysis was performed using One-Way-ANOVA and post-hoc (Newman Keuls) test. Statistical significance p ≤ 0.05 is considered.

## Results

Heparin was able to modify the inflammatory response of both cell populations, decreasing it significantly (p ≤ 0.05) in the case of macrophages (iNOS: Control:1 ± 0.09, Injured group:41.68 ± 4.86, Heparin group:0.54 ± 0.06. IL12p40: Control:1 ± 0.11, Injured group:46.74 ± 4.32, Heparin group:0.15 ± 0.009). The permeability of the monolayer and cell proliferation of alveolar cells did not show changes.

## Conclusions

Heparin has an immunomodulatory effect in alveolar cells and reduces inflammation in macrophages. This mechanism produced by heparin could have a beneficial effect in ALI.
